# High Leaf Vein Density Promotes Leaf Gas Exchange by Enhancing Leaf Hydraulic Conductance in *Oryza sativa* L. Plants

**DOI:** 10.3389/fpls.2021.693815

**Published:** 2021-10-25

**Authors:** Miao Ye, Meng Wu, Hao Zhang, Zuolin Zhang, Zujian Zhang

**Affiliations:** ^1^Key Laboratory of Crop Genetics and Physiology of Jiangsu Province, Jiangsu Key Laboratory of Crop Cultivation and Physiology, Co-innovation Center for Modern Production Technology of Grain Crops, Yangzhou University, Yangzhou, China; ^2^Ministry of Agriculture Key Laboratory of Crop Ecophysiology and Farming System in the Middle Reaches of the Yangtze River, College of Plant Science and Technology, Huazhong Agricultural University, Wuhan, China

**Keywords:** cultivated rice (*Oryza sativa* L.), leaf gas exchange, stomatal conductance, leaf hydraulics, leaf vein density

## Abstract

Six cultivated rice genotypes showing different stomatal conductance (*g*_s_) values were used to investigate the influence of leaf vein traits on leaf gas exchange and leaf hydraulics. The results showed that *g*_s_ was the main determinant of the varietal difference in the net photosynthetic rate (*P*_N_), whereas the area-based leaf nitrogen content (N_area_) and mesophyll conductance (*g*_m_) were not main factors. *g*_s_ and *P*_N_ were both positively correlated with leaf hydraulic conductance (*K*_leaf_). A high density of leaf veins (vein length per leaf area, VLA), especially minor leaf veins (VLA_minor_), was of benefit for improving the *K*_leaf_. The proportion of the minor leaf vein length to the total leaf vein length did not impact the leaf hydraulics or leaf gas exchange. Overall, these findings suggested that a high density of leaf veins, especially minor leaf veins, enhances *K*_leaf_ and promotes *g*_s_ and *P*_N_ in cultivated rice genotypes and a high VLA can be regarded as a high photosynthetic capacity trait in rice plants.

## Introduction

Under the current ambient atmospheric conditions, CO_2_ diffusional conductance from air to carboxylation sites is regarded as one of the main limiting factors of net photosynthetic rate (*P*_N_) in C_3_ plants (Evans et al., 2009; Li et al., 2009; Yamori et al., 2011; Flexas et al., 2012; Adachi et al., 2013; Gago et al., 2019). To reach carboxylation sites, CO_2_ in the air must first overcome the air–leaf boundary resistance to reach the surroundings of the stomata, and it then enters the stomatal pores to reach the substomatal cavity, diffuses to the surroundings of the cell wall, and successively passes through the cell wall, cell membrane, cytoplasm, chloroplast envelope, and the stroma (Terashima et al., 2011; Tholen et al., 2012). The CO_2_ diffusional resistance from air to the surface of the leaf is called boundary layer resistance, the CO_2_ diffusional resistance from air to the substomatal cavity is called stomatal resistance, and the CO_2_ diffusional resistance from the substomatal cavity to the carboxylation sites is called mesophyll resistance. Mesophyll resistance can be as important as stomatal resistance under many conditions (Terashima et al., 2011), although it was ignored in earlier studies (Farquhar et al., 1980; Kodama et al., 2011). The reciprocals of stomatal resistance and mesophyll resistance are called stomatal conductance (*g*_s_) and mesophyll conductance (*g*_m_), respectively. Many previous studies have shown that *P*_N_ is positively correlated with both *g*_s_ and *g*_m_ (Giuliani et al., 2013; Carriquí et al., 2015; Liu and Li, 2016).

Stomatal pores are the common pathway for CO_2_ entering the leaf and H_2_O evaporating from the leaf. *g*_s_ is mainly determined by the stomatal size, stomatal density and distribution, and especially by the stomatal aperture (Xu and Zhou, 2008; Ocheltree et al., 2012; Ouyang et al., 2017). *g*_s_ has been found to be closely linked to plant hydraulics (Hirasawa et al., 1992; Brodribb et al., 2007; Xiong and Nadal, 2020). Tabassum et al. (2016) reported that the *P*_N_, *g*_s_, and the transpiration rate (*E*) values in *Oryza sativa* L. plants were all positively correlated with whole-plant hydraulic conductance.

As leaf hydraulic conductance (*K*_leaf_) is one of the key components of whole-plant hydraulic conductance (Sack et al., 2003; Sack and Holbrook, 2006), it is vitally important for determining leaf gas exchange parameters. Brodribb et al. (2007) found that *P*_N_ was positively correlated with *K*_leaf_ across diverse terrestrial plants. Hirasawa et al. (1992) found that *g*_s_ was positively correlated with *K*_leaf_ in rice plants across different light and chemical treatments. Taylaran et al. (2011) found that the *P*_N_ of the high-yield rice cultivar Takanari was higher than that of the other common rice cultivars, which was partly due to its higher *K*_leaf_. In higher plants, transpiration drives water from the stem into the petiole, and then, the water enters the midrib, flows in an orderly fashion to different vein branches, passes through the vascular bundle sheath, enters the mesophyll tissue, evaporates into the intercellular airspace, and finally diffuses out of the leaf from stomatal pores (Rockwell et al., 2014; Xiong et al., 2017). Thus, leaf hydraulic transport can be divided into xylem hydraulic transport and outside-xylem hydraulic transport.

According to the water transport route inside leaves, *K*_leaf_ is mainly determined by the leaf vein traits such as the leaf vein density, the size of the xylem conduits within the bundle sheath (Flexas et al., 2013), and the leaf anatomical traits such as the fraction of intercellular airspace (Xiong et al., 2017). Many recent studies have focused on the relationships between *K*_leaf_ and leaf vein density (vein length per leaf area, VLA) (Xiong et al., 2015; Tabassum et al., 2016). Although a few studies have demonstrated that *K*_leaf_ showed no or even negative relationships with VLA, most studies illustrated that a high VLA is beneficial for improving *K*_leaf_ and promoting *g*_s_ and *P*_N_ (Sack and Frole, 2006; Brodribb et al., 2007; Boyce et al., 2009; Brodribb and Field, 2010; McKown et al., 2010; Field et al., 2011; Walls, 2011; Nardini et al., 2012; Flexas et al., 2013). High VLA can improve the parallel transport pathways per leaf area, thus improving the xylem hydraulic conductance (*K*_x_), and it can also shorten the distance from veins to evaporating sites, thus improving the outside-xylem hydraulic conductance (*K*_ox_) (Sack and Frole, 2006; Brodribb et al., 2007; Sack et al., 2013; Buckley et al., 2015).

Nevertheless, VLA was found to affect *K*_x_ but not *K*_ox_ or *K*_leaf_ across *Oryza* species, including cultivated and wild genotypes (Xiong et al., 2015, 2017). The irrelevance of *K*_ox_ and *K*_leaf_ with VLA may be due to the low ratio of xylem hydraulic resistance (*R*_x_) to leaf hydraulic resistance (*R*_leaf_), which is only 40% (Xiong et al., 2017). Thus, although a high VLA improved *K*_x_, the improvement in *K*_x_ contributed to *K*_leaf_ very slightly. Although the average *R*_x_/*R*_leaf_ in *Oryza* species was only 40%, the ratios in cultivated rice genotypes were much higher than 40% (Xiong et al., 2017), which may be due to the increase in VLA during domestication. Thus, although no correlations were found between *K*_leaf_ and VLA across *Oryza* species, these correlations may exist when only considering cultivated rice genotypes because cultivated genotypes have relatively high *R*_x_/*R*_leaf_ (Xiong et al., 2017). Therefore, we hypothesized that in cultivated rice genotypes in which *R*_x_/*R*_leaf_ is relatively high, high VLA can enhance *K*_leaf_ and promote leaf gas exchange.

In fact, the correlations among leaf gas exchange, leaf hydraulics, and leaf vein traits have already attracted a lot of attention. For example, Brodribb et al. (2007) reported that leaf maximum photosynthetic rate and venation are linked by hydraulics across 43 species. Brocious and Hacke (2016) found that stomatal conductance was positively correlated with the transport capacity of the petiole, estimated from the diameter and number of xylem vessels, whereas the variation in stomatal conductance and leaf hydraulic conductance was not linked to vein density or other anatomical lamina properties across different *populus* genotypes. However, most previous studies were done in wood species, and rare studies were done in cereal species, especially in rice.

In the present study, six rice genotypes that showed different *g*_s_ values in a previous study (Ye et al., 2019) were selected to verify the hypothesis that high VLA can enhance *K*_leaf_ and promote leaf gas exchange in cultivated rice genotypes. The results may provide insights for high photosynthetic capacity rice breeding.

## Materials and Methods

### Plant Materials

A pot experiment was conducted at the Huazhong Agricultural University (114.37°E, 30.48°N), Wuhan, Hubei Province, China. Six cultivated rice genotypes (*O. sativa* L.) found worldwide were used, including Kirmizi Celtik, Huayou 675, Teqing, Huanghuazhan, Champa, and N22. The *g*_s_ of these six genotypes varied greatly in the study by Ye et al. (2019), thus indicating feasibility for studying the underlying mechanisms for different *g*_s_ and *P*_N_ values in rice. Rice plants were grown from September to December 2014. After germination on moist filters, seeds were transferred to nursery plates. When the seedlings had developed an average of three leaves, they were transplanted to 11 L pots with a density of three hills per pot and two seedlings per hill. There were five pots per genotype, and each pot was filled with 10 kg of soil. The soil used for the experiment had a clay loam texture, with pH 6.63, organic matter 6.42 g/kg, total nitrogen (N) 0.07 mg/kg, available phosphorus (P) 8.21 mg/kg, and available potassium (K) 126.95 mg/kg. P and K were applied as basal fertilizers at an amount of 1.5 g/pot. N was applied at an amount of 2.0 g(N)/pot, with 40% applied as a basal fertilizer, and 60% applied at the mid-tillering stage. Plants were watered daily, and a minimum 2 cm water layer was maintained to avoid drought stress. Pests were intensively controlled using chemical pesticides.

Rice plants were grown outdoors. All genotypes were arranged in a random design with five replicates. The radiation intensity, average temperature, and relative humidity during rice growth were 12.6 ± 5.7 MJ/m^2^/d, 23.3 ± 3.4°C, and 74.4 ± 10.4%, respectively. Gas exchange measurements were conducted in a growth chamber (Conviron GR48, Controlled Environments Limited, Winnipeg, MB, Canada) [photosynthetic photon flux density (PPFD), 1,000 μmol(photo)/m^2^/s at the leaf level; temperature, 28°C; relative humidity, 60%; and CO_2_ concentration, 400 μmol/mol] to avoid the influence of a changing environment on gas exchange parameters. The vapor pressure deficit between leaf and air (VPD_leaf−air_) of the six rice genotypes during the measurements is shown in Supplementary Table 1. The measurements were conducted 2 weeks after mid-tillering fertilization, and all measurements were conducted on the newly expanded leaves from three different pots of each genotype.

### Gas Exchange and Chlorophyll Fluorescence Measurements

A portable photosynthesis system (LI-6400XT, LI-CORInc., Lincoln, NE, United States) with an integrated fluorescence leaf chamber (Li-6400-40; Li-Cor) was used to measure gas exchange and chlorophyll fluorescence on leaves between 08:00 and 16:00. Measurements began after the plants had acclimatized to the chamber for ~2 h. In the LI-6400XT cuvette, the ambient CO_2_ concentration was controlled and set to 400 μmol/mol, the leaf temperature was maintained at 28°C, the PPFD was 1,500 μmol(photo)/m^2^/s, and the flow rate was 500 μmol/s. After reaching a steady state, which usually takes 25 min, the gas exchange parameters, steady-state fluorescence (*F*_s_), and maximum fluorescence (*F*_m_') were recorded with a light saturating pulse of 8,000 μmol(photo)/m^2^/s. The actual photochemical efficiency of photosystem II (Φ_PSII_) was calculated as follows:


(1) $$\Phi_{\text{PSII}}=\frac{\left(F_{m}\prime -F_{s}\right)}{F_{m}\prime }$$

The electron transport rate (*J*) was calculated as follows:


(2) $$\text{J}=\text{PPFD}\times \alpha \beta \times \Phi_{\text{PSII}}$$

where α is the leaf absorptance and β is the partitioning of absorbed quanta between photosystem II and photosystem I. The product αβ was determined from the slope of the relationship between Φ_PSII_ and the quantum efficiency of CO_2_ uptake (Φ_CO2_), which was obtained by varying the light intensity under non-photorespiratory conditions at <2% O_2_ (Valentini et al., 1995).

The variable *J* method described by Harley et al. (1992) was used to calculate the chloroplastic CO_2_ concentration (*C*_c_) and *g*_m_. *C*_c_ and *g*_m_ were calculated as follows:


(3) $$C_{c}=\frac{\Gamma^{*}\times [J+8\times (P_{N}+R_{d})]}{J-4\times (P_{N}+R_{d})}$$

(4) $$g_{m}=\frac{P_{N}}{C_{i}-C_{c}}$$

where Γ* represents the CO_2_ compensation point in chloroplasts without day respiration. The day respiration (*R*_d_) and the apparent CO_2_ photocompensation point ($C_{\text{i}}^{*}$) were determined using the Laisk method (Brooks and Farquhar, 1985). Briefly, *A*/*C*_i_ (*A*, net photosynthetic rate; *C*_i_, intercellular CO_2_ concentration) curves were measured over the linear portion of the response curve (at 100, 80, 50, and 25 μmol CO_2_/mol air) over three PPFDs (150, 300, and 600 μmol/m^2^/s) with an LI 6400-02B chamber (Li-Cor), and then linear regressions to the responses for each PPFD were fitted for individual leaves. The intersection point of three *A*/*C*_i_ curves was considered as $C_{\text{i}}^{*}$ (*x*-axis) and *R*_d_ (*y*-axis) (von Caemmerer et al., 1994). Γ* was calculated as follows:


(5) $$\Gamma^{*}=C_{i}^{*}+R_{\text{d}}/g_{\text{m}}$$

The Γ* and *R*_d_ of the six rice genotypes are shown in Supplementary Table 2.

### Leaf Hydraulic Conductance

The evaporative flux method (EFM) was used to determine the *K*_leaf_ (Sack et al., 2002; Brodribb et al., 2007; Guyot et al., 2012; Sack and Scoffoni, 2012; Tabassum et al., 2016). A leaf of each genotype was excised in water, and the base of the leaf was placed in a test tube filled with distilled water under favorable conditions for transpiration (in a growth chamber under a PPFD of 1,000 μmol(photo)/m^2^/s and air temperature of 28°C). Immediately after placing the leaf in a test tube filled with distilled water, the leaf was attached to a Li-COR 6400XT portable infrared gas analyzer (IRGA) (LI-COR, NE, United States) to record the leaf transpiration rate (*E*). A Dewpoint Potential Meter WP4C (Decagon, Pullman, WA, United States) was used to measure the leaf water potential (Ψ_leaf_) of the leaf that was used to measure *E*. *K*_leaf_ was calculated as follows (Taylaran et al., 2011):


(6) $$K_{leaf}=\frac{E}{0-\Psi_{leaf}}$$

### Leaf Morphological Traits

Leaf width was measured *in vivo*, and leaves were excised to count the major veins (midrib vein and large veins) and minor veins separately under 40× magnification using a light microscope (SA3300, Beijing Tech Instrument Co., Ltd, Beijing, China). Four leaves were counted for each genotype. As rice leaf veins are parallel to each other, the major leaf vein density (VLA_major_), minor leaf vein density (VLA_minor_), and total leaf vein density (VLA) were calculated as follows:


(7) $${\text{VLA}}_{\text{major}}=\frac{\text{Numbers of leaf major veins}\times \text{leaf Length}}{\text{Leaf width}\times \text{leaf length}}$$

(8) $${\text{VLA}}_{\text{minor}}=\frac{\text{Numbers of leaf minor veins}\times \text{leaf Length}}{\text{Leaf width}\times \text{leaf length}}$$

(9) $$\text{VLA}={\text{VLA}}_{\text{major}}+{\text{VLA}}_{\text{minor}}$$

### Leaf Nitrogen Content

The leaf used for gas exchange measurements was detached, and another two leaves were also detached, then the leaf area of these three leaves was measured using a LI-Cor 3000C (LI-COR Inc., Lincoln, NE, United States) leaf area analyzer. Leaves were then oven-dried at 80°C until they achieved a constant weight. Afterward, the leaf dry mass was weighed and the leaf mass per area (LMA) was calculated as the ratio of the leaf dry mass to leaf area. The leaf N content based on leaf mass (N_mass_, %) was measured with an Elementar Vario MAX CN analyzer (Elementar Analysesysteme GmbH, Hanau, Germany), and the area-based leaf N content (N_area_) was calculated by multiplying N_mass_ with LMA.

### Statistical Analysis

One-way analysis of variance (ANOVA) was used to assess the effects of the genotypes on each parameter using Statistix 9.0 software (Analytical Software, Tallahassee, FL, United States). Parameters were compared between genotypes based on the least significant difference (LSD) test level at the 0.05 probability level. Graphs were created, and a linear regression analysis was performed to test the correlations between the parameters using SigmaPlot 10.0 (Systat Software Inc., CA, United States).

## Results

### Varietal Differences in Leaf Gas Exchange Parameters and N_area_

**Figure 1:**
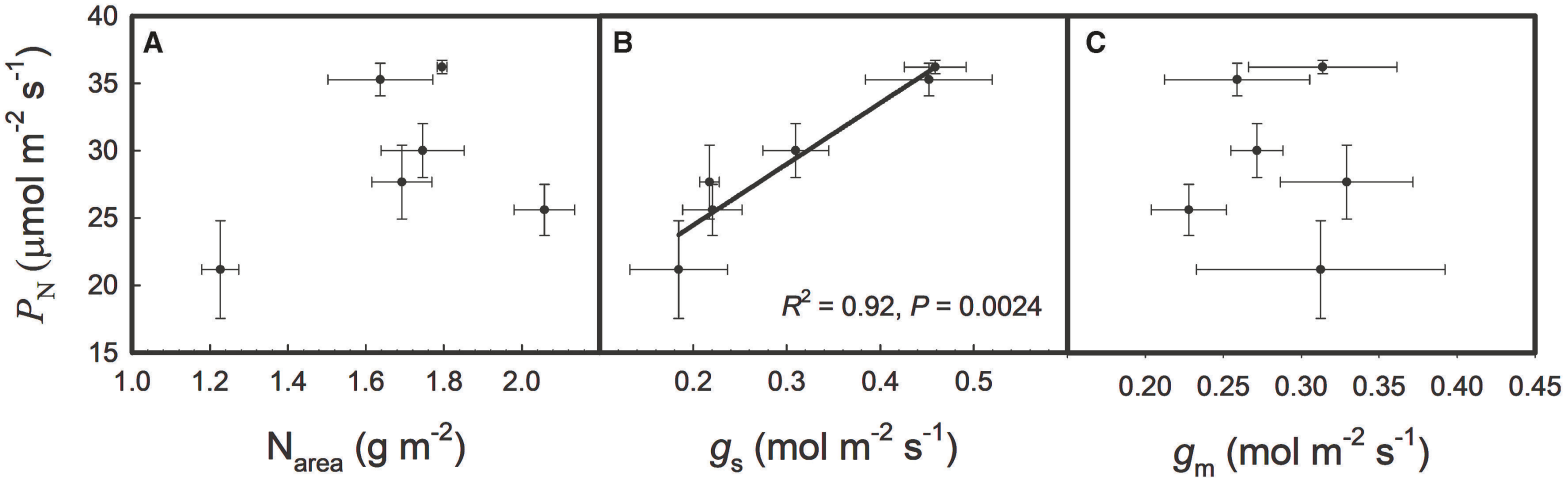
Relationships of net photosynthetic rate (P_N_) with: **(A)** area-based leaf nitrogen content (N_area_), **(B)** stomatal conductance (g_s_), and **(C)** mesophyll conductance (g_m_) across the six rice genotypes. Data are shown as the means ± SD of three replicates.

As shown in Table 1, P_N_, N_area_, and g_s_ showed significant varietal differences among the six rice genotypes. P_N_ ranged from 21.2 to 36.2 μmol/m^2^/s, and the highest and the lowest values were found in Champa and N22, respectively. Kirmizi Celtik showed the highest N_area_ of 2.03 g/m^2^ and N22 showed the lowest N_area_ of 1.23 g/m^2^. g_s_ ranged from 0.18 to 0.46 mol/m^2^/s and the highest and lowest values were found in Champa and N22, respectively. g_m_ showed no significant varietal difference among the six rice genotypes. The highest g_m_ was 0.33 mol/m^2^/s, which was found in Teqing, whereas the lowest g_m_ was 0.23 mol/m^2^/s, which was found in Kirmizi Celtik. As shown in Figure 1, P_N_ was not correlated with N_area_ or g_m_ but was positively correlated with g_s_ across the six rice genotypes.

**Table 1:** Gas exchange parameters and area-based leaf nitrogen content (N_area_) of the six rice genotypes.

**Genotype**	***P*_**N**_ (μmol/m^**2**^/s)**	**N_**area**_** **(g/m^**2**^)**	***g*_**s**_ (mol/m^**2**^/s)**	***g*_**m**_ (mol/m^**2**^/s)**
Champa	36.2 ± 0.5 a	1.80 ± 0.01 b	0.46 ± 0.03 a	0.31 ± 0.05 a
Huayou 675	35.3 ± 1.2 a	1.64 ± 0.13 b	0.45 ± 0.07 a	0.26 ± 0.05 ab
Teqing	27.7 ± 2.7 bc	1.69 ± 0.08 b	0.22 ± 0.01 c	0.33 ± 0.04 a
Huanghuazhan	30.0 ± 2.0 b	1.75 ± 0.11 b	0.31 ± 0.04 b	0.27 ± 0.02 ab
Kirmizi Celtik	25.6 ± 1.9 c	2.06 ± 0.08 a	0.22 ± 0.03 c	0.23 ± 0.02 b
N22	21.2 ± 3.6 d	1.23 ± 0.05 c	0.18 ± 0.05 c	0.31 ± 0.08 a
ANOVA				
Average	29.3	1.69	0.31	0.29
Genotype	^***^	^***^	^***^	ns

*Data are shown as the means ± SD of three replicates*.

*** *indicates significance at the 0.001 level; ns indicates not significant at the 0.05 level. Within a column, different letters represent data that are significantly different from each other in the LSD (0.05)*.

### Varietal Differences in the Leaf Morphological Traits and Leaf Hydraulics

As shown in Table 2, leaf width showed significant varietal differences. Champa possessed the widest leaf of 14.2 mm, whereas Kirmizi Celtik possessed the narrowest leaf of 9.4 mm. VLA, VLA_minor_, and the proportion of minor leaf vein length to the total leaf vein length all showed no significant varietal differences. VLA_major_ showed significant varietal differences. Huayou 675 had the largest VLA_major_ of 0.82 mm/mm^2^, and N22 had the smallest VLA_major_ of 0.66 mm/mm^2^. *E*, Ψ_leaf_, and *K*_leaf_ all showed no significant varietal differences.

**Table 2:** Leaf morphological traits and leaf hydraulics of the six rice genotypes.

**Genotype**	**Leaf width (mm)**	**VLA (mm/ mm^**2**^)**	**VLA_**major**_ (mm/mm^**2**^)**	**VLA_**minor**_ (mm/mm^**2**^)**	**Proportion of minor leaf vein length to total leaf vein length (%)**	***E* (mmol/m^**2**^/s)**	**Ψ_**leaf**_ (MPa)**	***K*_**leaf**_ (mmol/m^**2**^/s/MPa)**
Champa	14.2 ± 0.8 a	4.98 ± 0.19 a	0.81 ± 0.09 a	4.16 ± 0.17 a	83.6 ± 1.5 a	8.95 ± 0.21 a	−0.69 ± 0.03 a	13.0 ± 0.9 a
Huayou 675	11.2 ± 0.1 cd	4.94 ± 0.32 ab	0.82 ± 0.05 a	4.11 ± 0.36 a	83.2 ± 2.2 a	8.31 ± 1.90 a	−0.64 ± 0.09 a	12.9 ± 2.6 a
Teqing	13.4 ± 2.7 ab	4.58 ± 0.46 ab	0.80 ± 0.13 a	3.77 ± 0.38 a	82.5 ± 1.9 a	7.66 ± 1.28 a	−0.73 ± 0.22 a	10.6 ± 1.8 ab
Huanghuazhan	10.8 ± 1.2 cd	4.55 ± 0.21 ab	0.72 ± 0.05 ab	3.83 ± 0.20 a	84.1 ± 1.0 a	8.04 ± 1.09 a	−0.68 ± 0.03 a	11.7 ± 1.1 ab
Kirmizi Celtik	9.4 ± 0.8 d	4.34 ± 0.83 b	0.72 ± 0.09 ab	3.62 ± 0.82 a	82.9 ± 3.8 a	7.05 ± 0.93 a	−0.74 ± 0.15 a	9.6 ± 1.2 b
N22	11.8 ± 1.5 bc	4.32 ± 0.14 b	0.66 ± 0.04 b	3.66 ± 0.14 a	84.7 ± 0.9 a	7.86 ± 2.30 a	−0.81 ± 0.09 a	9.7 ± 2.4 b
ANOVA								
Average	11.8	4.62	0.76	3.86	83.5	7.98	−0.71	11.3
Genotype	^**^	ns	^*^	ns	ns	ns	ns	ns

*Data are shown as the means ± SD. There were four replicates for leaf morphological traits and three replicates for E, Ψ_leaf_, and K_leaf_*.

* and ** *indicate significance at the 0.05 and 0.01 levels, respectively; ns indicates non-significant at the 0.05 level. Within a column, different letters represent data that are significantly different from each other in the LSD (0.05). VLA, vein length per leaf area*.

### Relationships Among the Leaf Gas Exchange Parameters, Leaf Hydraulics, and Leaf Morphological Traits

**Figure 2:**
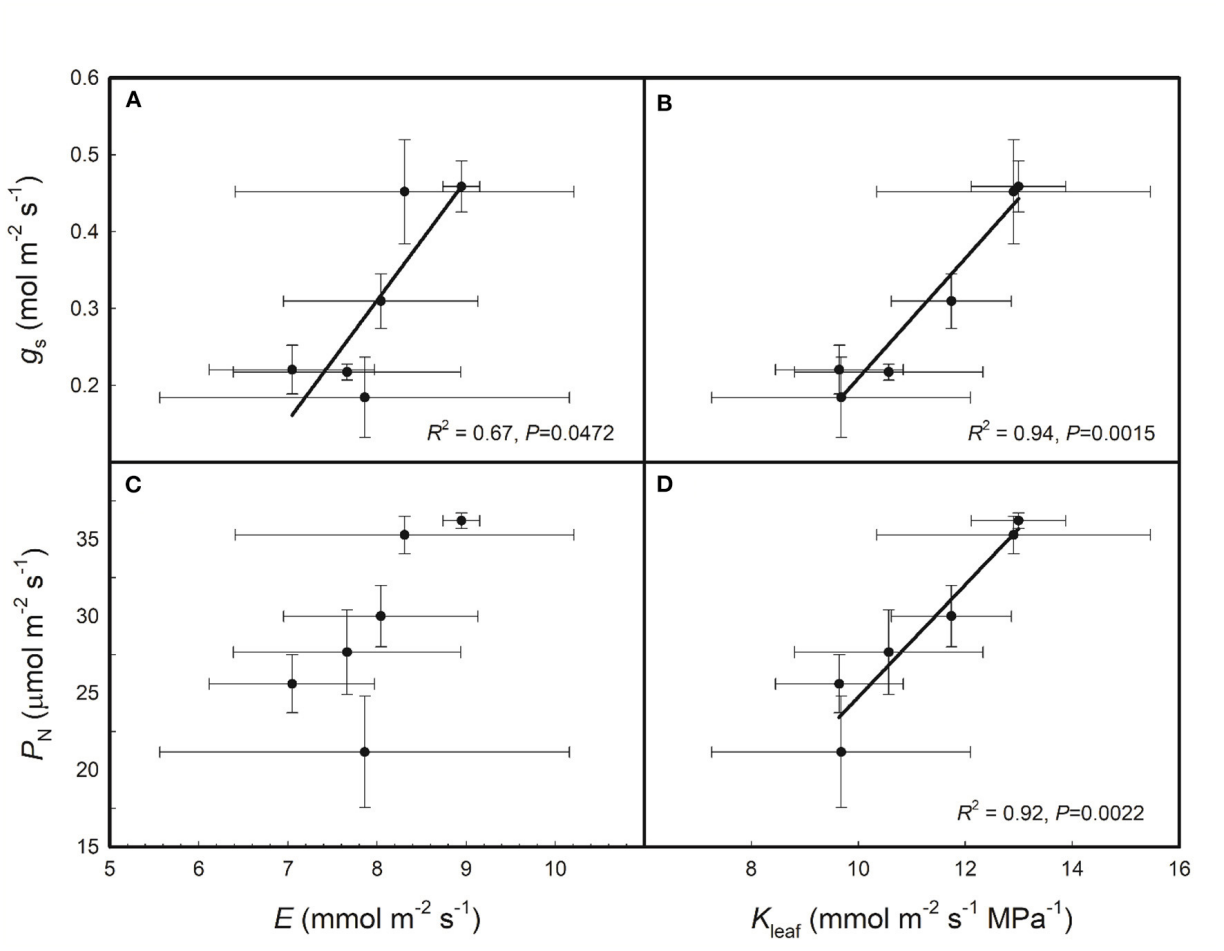
Relationships of stomatal conductance (*g*_s_) and net photosynthetic rate (*P*_N_) with leaf transpiration rate (*E*) **(A,C)** and leaf hydraulic conductance (*K*_leaf_) **(B,D)** across the six rice genotypes. Data are shown as the means ± SD of three replicates.

**Figure 3:**
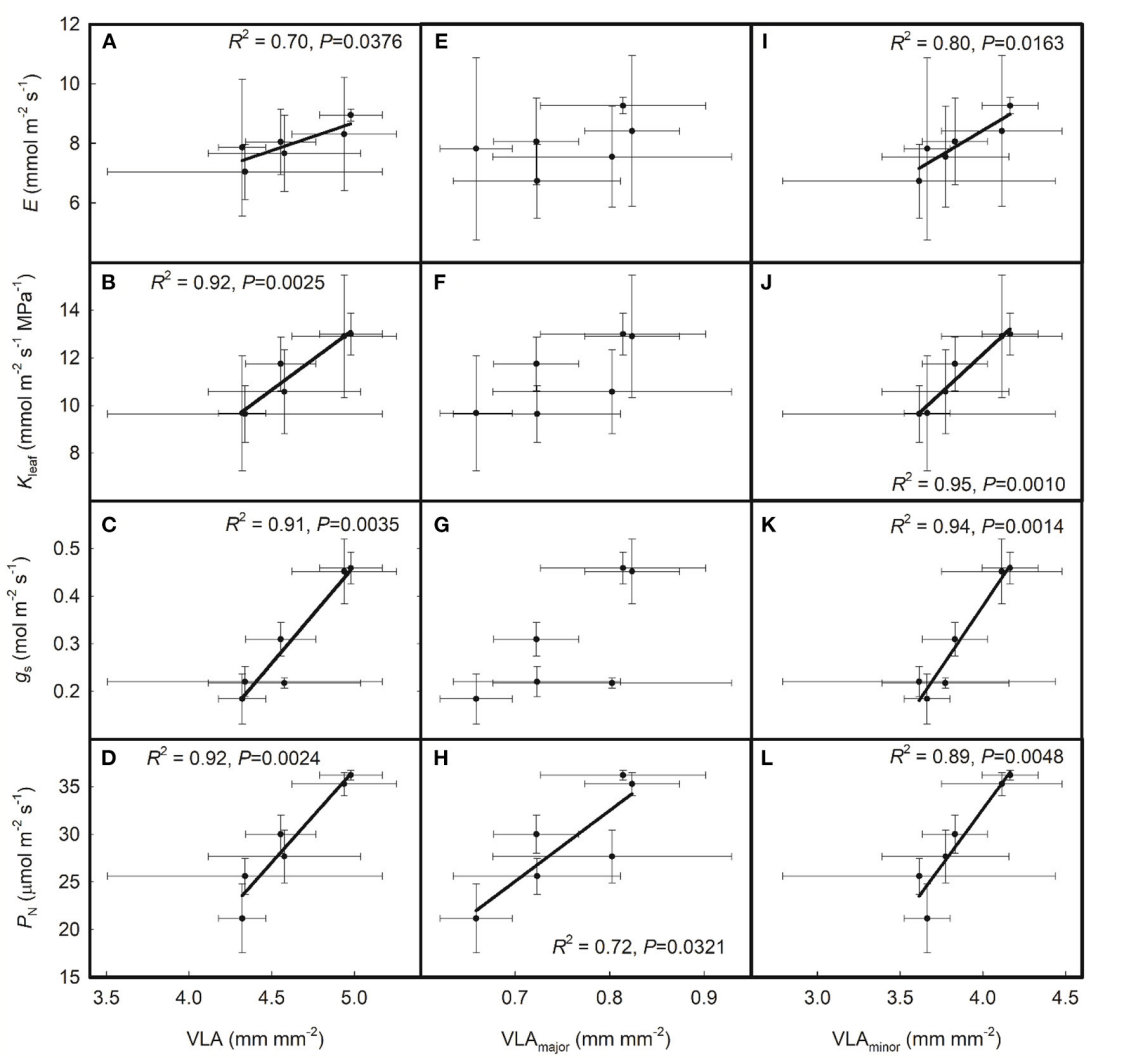
Relationships of the leaf hydraulics and leaf gas exchange parameters with **(A–D)** total leaf vein density (VLA), **(E–H)** major leaf vein density (VLA_minor_), and **(I–L)** minor leaf vein density (VLA_minor_) across the six rice genotypes. Data are shown as the means ± SD. There were four replicates for leaf vein densities and three replicates for P_N_, g_s_, K_leaf_, and E.

As shown in Figure 2, g_s_ was positively correlated with both E and K_leaf_ across the six rice genotypes and P_N_ was positively correlated with K_leaf_ but had no relationship with E across the six rice genotypes. As shown in Figure 3, E, K_leaf_, g_s_, and P_N_ were all positively correlated with VLA and VLA_minor_ across the six rice genotypes; P_N_ was positively correlated with VLA_major_, but E, K_leaf_, and g_s_ had no relationship with VLA_major_ across the six rice genotypes.

## Discussion

In the present study, *P*_N_ was not correlated with N_area_ and *g*_m_ but was correlated with *g*_s_ and *K*_leaf_. VLA and VLA_minor_ showed no varietal differences in the present study, which was perhaps due to the few genotypes used, and their vein traits ranged very narrowly. Rice leaf veins are distributed in parallel, and the leaf vein density is strongly affected by the leaf width (Baird et al., 2021). For example, Tabassum et al. (2016) reported that polyethylene glycol–induced water deficit caused rice leaf narrowing and led to an increase in leaf density. Xiong et al. (2015) investigated the leaf morphological traits of 11 rice genotypes, including seven wild genotypes and four cultivated genotypes, and found that leaf morphological traits showed great varietal differences because the leaf area showed ~seven times the variation, ranging from 18.4 to 127.3 cm^2^, and leaf width showed ~six times the variation, ranging from 0.38 to 2.20 cm. The leaf width in the present study only showed 1.5 times the variation, ranging from 9.4 to 14.2 mm. Thus, leaf vein densities in the present study did not vary greatly. More genotypes and varying environmental conditions should be included in future studies to investigate the varietal differences in rice leaf vein traits and their impacts on leaf gas exchange.

In the present study, *K*_leaf_, *E, g*_s_, and *P*_N_ all substantially increased with VLA and VLA_minor_ across the six cultivated rice genotypes, thus verifying the hypothesis that high VLA can enhance *K*_leaf_ and promote leaf gas exchange in cultivated rice genotypes in which *R*_x_/*R*_leaf_ is relatively high. By sorting the correlations between leaf gas exchange parameters, leaf hydraulic parameters, and leaf vein densities, we drew the conclusion that leaf vein density affected leaf gas exchange parameters by regulating leaf water transport. However, we still lack the analyses of many other vein traits such as the diameter of xylem conduits and the outside xylem anatomical traits on leaf hydraulics and gas exchange. More studies should be done to explore the coordination between the physiological and structural traits of the leaf.

**Figure 4:**
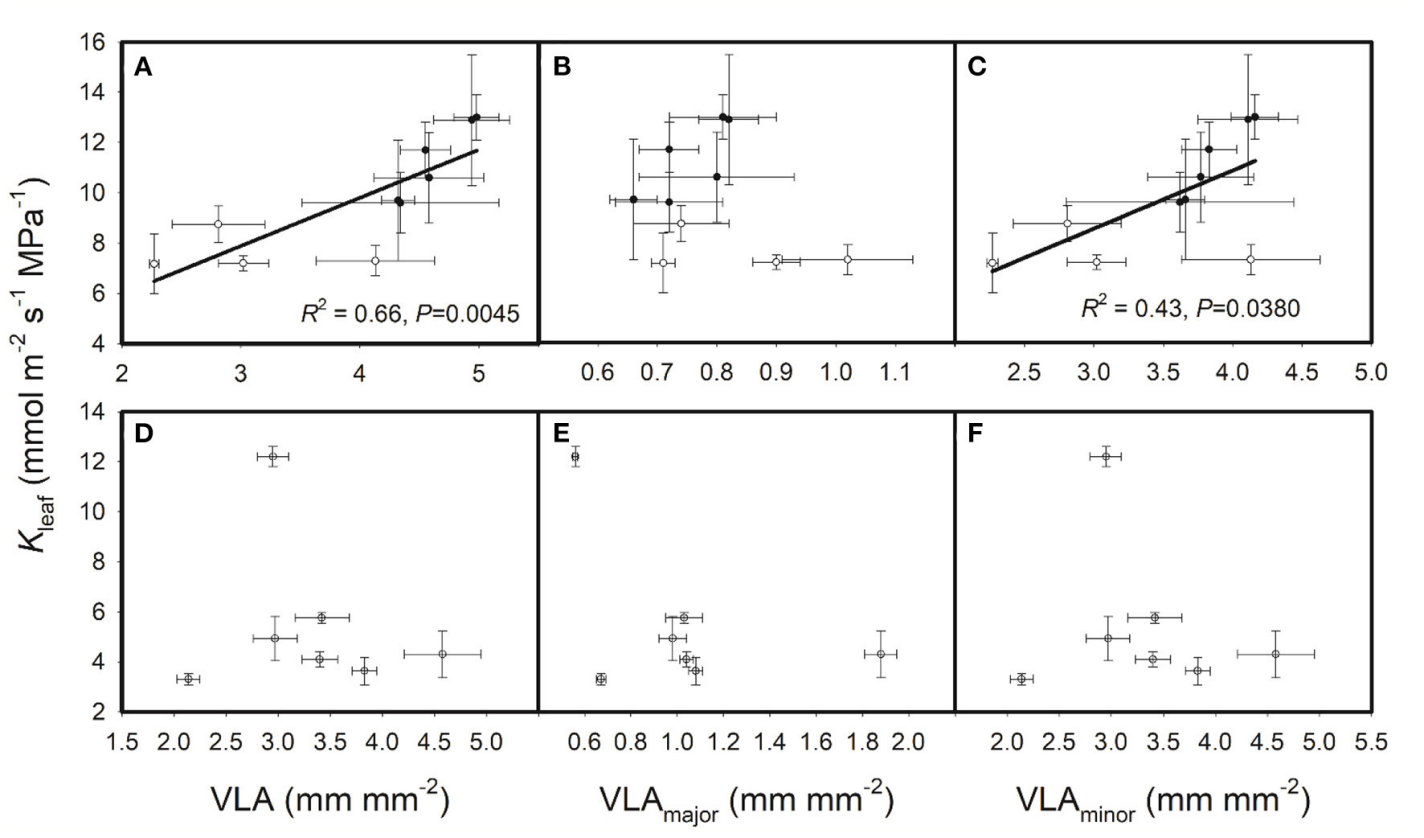
Relationships of leaf hydraulic conductance (K_leaf_) with leaf vein densities across **(A–C)** cultivated rice genotypes and **(D–F)** wild rice genotypes. The solid circles are data for the six cultivated rice genotypes in the present study, and the empty circles are data from Xiong et al. (2015, 2017) studies.

Furthermore, VLA and *K*_leaf_ data from other studies (Xiong et al., 2015, 2017) were extracted to verify our hypothesis. As shown in Figure 4, *K*_leaf_ was positively correlated with VLA and VLA_minor_ across the cultivated rice genotypes, whereas these correlations disappeared across the wild rice genotypes. Therefore, we conclude that in cultivated rice genotypes, high VLA and high VLA_minor_ can enhance *K*_leaf_ and promote leaf gas exchange, and a high VLA can be regarded as a feature of rice materials having high photosynthetic capacity. Variations in VLA drove the correlation between *K*_leaf_ and VLA, which was probably due to the determining role of VLA on *K*_leaf_, as other features of hydraulic architecture may vary slightly in cultivated rice genotypes. However, this correlation may be obscured when other features of hydraulic architecture vary. For example, the positive effects of high VLA on *K*_leaf_ may be attenuated by the negative effects of small xylem conduits in a plant. Besides, as one of the few studies on cereal crops, the present study showed different results with previous studies on wood species, for example, *populus* plants in Brocious and Hacke's (Brocious and Hacke, 2016) study. A positive correlation was found between *K*_leaf_ and VLA in the present study but not in Brocious and Hacke (2016) may also be attributed to the different importance of VLA to *K*_leaf_ in the species. The comprehensive effects of hydraulic architecture on leaf hydraulics should be summarized.

Correlations between *K*_leaf_ and VLA existing in cultivated but not wild rice genotypes illustrate the importance of leaf vein traits during rice domestication or genetic improvement. For example, Wu et al. (2020) revealed that an increase in leaf vein dimensions during rice genetic improvement enhanced leaf gas exchange. In addition to leaf vein traits, leaf morphological traits associated with leaf gas exchange, such as stomatal density and stomatal size in rice, have also been improved during crop genetic improvement (Panda et al., 2018; Wu et al., 2020). There is a need for establishing the underlying morphological and physiological changes for crop improvement.

A previous study (Venturas et al., 2017) illustrated that minor leaf vein length accounts for more than 80% of the total leaf vein length in higher plants. In the present study, minor leaf vein length accounted for 83.5% of the total leaf vein length on average (Table 2). Xiong et al. (2015) reported that the proportion of minor leaf vein length to total leaf vein length but not leaf vein density is the critical factor determining *K*_leaf_. However, in the present study, neither *E, K*_leaf_, *g*_s_, nor *P*_N_ showed significant correlations with the proportion of minor leaf vein length to total leaf vein length (data not shown).

Except for the leaf vein density, other vein traits also greatly impacted *K*_leaf_. Previous studies documented that short distance from minor veins to stomata and large diameters of xylem conduit accompanied by thin xylem conduit walls were beneficial for improving *K*_leaf_ (Brodribb et al., 2007; Sack and Scoffoni, 2013; Venturas et al., 2017; Xiong and Nadal, 2020). Nardini et al. (2014) found that large leaves tend to have higher VLA_major_ than small leaves and can promote *K*_leaf_. Simonin et al. (2012) demonstrated that *K*_leaf_ increased with leaf area. However, a recent study exploring the development and biophysical determinations of grass leaf size worldwide revealed that small leaves have hydraulic benefits due to their higher VLA_major_ (Baird et al., 2021). Optimal leaf morphological and anatomical traits for improving *K*_leaf_ and *P*_N_ should be defined to provide theories for high photosynthetic capacity rice breeding.

*K*_x_ is mainly determined by leaf vein traits, whereas *K*_ox_ is mainly determined by leaf anatomical traits. Xiong et al. (2017) found that *K*_ox_ is positively correlated with the fraction of intercellular airspace, the mesophyll cell surface area exposed to intercellular airspace per leaf area, and the surface area of chloroplasts exposed to intercellular airspace per leaf area, whereas it is negatively correlated with cell wall thickness. *g*_m_ has been reported to be positively correlated with *g*_s_ in recent years (Flexas et al., 2013). One reason for the positive relationship between *g*_s_ and *g*_m_ is that they are both positively correlated with *K*_leaf_ (Xiong and Nadal, 2020). *g*_s_ is positively correlated with *K*_leaf_ due to the close relationship between stomatal opening and leaf water transport; *g*_m_ is positively correlated with *K*_leaf_ and *K*_ox_ because *g*_m_ and *K*_ox_ are mediated by leaf anatomical traits (Xiong et al., 2017). However, *K*_x_, *K*_ox_, and leaf anatomical traits were not measured in the present study, and more studies should be done to investigate and explain the coordination among leaf gas exchange, leaf hydraulics, and leaf anatomy.

In addition, the water transport pathways inside leaves, i.e., where the evaporating sites occur, are currently unclear. Rockwell et al. (2014) reported that water dominantly evaporated from the vascular bundle sheath and its surroundings to the intercellular airspace in *Quercus rubra*. Moreover, the conversion of water between the vapor and liquid phases is unknown. For example, Buckley et al. (2015) found that vapor phase transport contributed 39.25–44.00% to *K*_ox_ when the temperature difference between the xylem and leaf epidermis reached 0.2 K, suggesting that temperature greatly affects leaf hydraulic status. Evaporating sites and leaf hydraulic status inside rice leaves need more investigation.

## Conclusions

In the present study, VLA_major_ showed great varietal differences among cultivated rice genotypes, whereas VLA, VLA_minor_, and the proportion of minor leaf vein length to total leaf vein length showed no varietal differences. *E, K*_leaf_, *g*_s_, and *P*_N_ were all positively correlated with VLA and VLA_minor_, and *P*_N_ was positively correlated with VLA_major_. We concluded that high VLA and high VLA_minor_ can enhance *K*_leaf_ and promote leaf gas exchange in the cultivated rice genotypes and high VLA can be regarded as a high photosynthetic capacity trait in rice plants.

## Data Availability Statement

The original contributions presented in the study are included in the article/Supplementary Material, further inquiries can be directed to the corresponding author.

## Author Contributions

MY designed the research and wrote the paper. MY and ZuoZ conducted the experiments. MY and MW analyzed the data. HZ and ZujZ commented and revised the paper. All authors contributed to the article and approved the submitted version.

## Funding

This research was funded by the National Natural Science Foundation of China, grant nos. 31871532 and 31871559, the National Key Research and Development Program of China, grant nos. 2016YFD0300102 and 2016YFD0300502, and the Priority Academic Program Development of Jiangsu Higher Education Institutions.

## Conflict of Interest

The authors declare that the research was conducted in the absence of any commercial or financial relationships that could be construed as a potential conflict of interest.

## Publisher's Note

All claims expressed in this article are solely those of the authors and do not necessarily represent those of their affiliated organizations, or those of the publisher, the editors and the reviewers. Any product that may be evaluated in this article, or claim that may be made by its manufacturer, is not guaranteed or endorsed by the publisher.
