# Supplementary material for: High Leaf Vein Density Promotes Leaf Gas Exchange by Enhancing Leaf Hydraulic Conductance in Oryza sativa L. Plants
Source: Front Plant Sci. 2021 Oct 25;12:693815. doi: 10.3389/fpls.2021.693815 (PMC8573028; doi:10.3389/fpls.2021.693815)
Supplement: Supplementary file 1 [file Data_Sheet_1.pdf]

Supplementary Table 1. The vapor pressure deficit between leaf and air ($VPD_{leaf\text{-}air}$) of the six rice genotypes. Data are shown as the means ± SD of three replicates. * indicates significance at the 0.05 levels. Within the column, different letters represent data that are significantly different from each other in the LSD (0.05).

Genotype	$VPD_{leaf\text{-}air}$ (MPa)
CHAMPA	1.39 ± 0.05 bc
Huayou 675	1.26 ± 0.05 c
Teqin	1.74 ± 0.27 a
Huanghuazhan	1.57 ± 0.13 ab
Kirmizi Celtik	1.70 ± 0.19 a
N22	1.69 ± 0.12 a
ANOVA	
Average	1.56
Genotype	*

Supplementary Table 2. The CO_2 compensation point in chloroplasts without day respiration (Γ^*) and the day respiration rate (R_d) of the six rice genotypes. Data are shown as the means ± SD of three replicates. *** indicates significance at the 0.001 level. Within a column, different letters represent data that are significantly different from each other in the LSD (0.05).

Genotype	Γ^* (μmol mol^{-1})	R_d (μmol m^{-2} s^{-1})
CHAMPA	44.08 ± 0.17 c	0.395 ± 0.053 bc
Huayou 675	41.72 ± 0.16 d	0.306 ± 0.028 c
Teqin	46.00 ± 0.24 b	0.533 ± 0.142 b
Huanghuazhan	42.51 ± 0.07 d	0.277 ± 0.163 c
Kirmizi Celtik	44.10 ± 0.23 c	0.441 ± 0.059 bc
N22	51.59 ± 1.25 a	1.350 ± 0.003 a
ANOVA		
Average	45.00	0.551
Genotype	***	***
